# Bisphenols—A Threat to the Natural Environment

**DOI:** 10.3390/ma16196500

**Published:** 2023-09-29

**Authors:** Magdalena Zaborowska, Jadwiga Wyszkowska, Agata Borowik, Jan Kucharski

**Affiliations:** Department of Soil Science and Microbiology, University of Warmia and Mazury in Olsztyn, Plac Łódzki 3, 10-727 Olsztyn, Poland; m.zaborowska@uwm.edu.pl (M.Z.); agata.borowik@uwm.edu.pl (A.B.); jan.kucharski@uwm.edu.pl (J.K.)

**Keywords:** bisphenol A, bisphenol F, bisphenol S, xenobiotics, microbiome, soil enzymes, soil microorganism, structural diversity, crops

## Abstract

Negative public sentiment built up around bisphenol A (BPA) follows growing awareness of the frequency of this chemical compound in the environment. The increase in air, water, and soil contamination by BPA has also generated the need to replace it with less toxic analogs, such as Bisphenol F (BPF) and Bisphenol S (BPS). However, due to the structural similarity of BPF and BPS to BPA, questions arise about the safety of their usage. The toxicity of BPA, BPF, and BPS towards humans and animals has been fairly well understood. The biodegradability potential of microorganisms towards each of these bisphenols is also widely recognized. However, the scale of their inhibitory pressure on soil microbiomes and soil enzyme activity has not been estimated. These parameters are extremely important in determining soil health, which in turn also influences plant growth and development. Therefore, in this manuscript, knowledge has been expanded and systematized regarding the differences in toxicity between BPA and its two analogs. In the context of the synthetic characterization of the effects of bisphenol permeation into the environment, the toxic impact of BPA, BPF, and BPS on the microbiological and biochemical parameters of soils was traced. The response of cultivated plants to their influence was also analyzed.

## 1. Introduction

The expanding production of a broad range of organic compounds, including bisphenols, aligns with and contributes to the dynamics of the Anthropocene epoch, resulting in disruptions to ecosystem equilibrium. The identification of sources, characteristics, and the scale of the annually increasing dispersion of bisphenol A (BPA) in the environment has contributed to the cultivation of negative societal sentiments surrounding this phenolic compound. This has led to the exploration of substitutes with potentially lower toxicity. Currently, among the 16 substitutes for BPA, bisphenol F (BPF) and bisphenol S (BPS) are the most commonly employed in the chemical industry [1].

The first scientific data on BPA were published in 1905 by Thomas Zincke from the University of Marburg in Germany. In 1957, commercial production of BPA began in the United States, followed by its initiation in Europe in 1958, coinciding with the increasing use of plastics [2]. The significant interest in BPA, particularly as a monomer, is linked to its ability to delay the oxidative degradation of plastics exposed to UV radiation [3]. It is also a constituent of polyesters and polyacrylates and serves as a stabilizer and antioxidant in PVC production [4,5]. However, its primary applications lie in the production of polycarbonate and epoxy resins, accounting for 65% and 30% of its utilization, respectively [6]. The widespread occurrence of BPA in the environment is attributed to polycarbonate materials being a fundamental component of electrical and electronic devices, safety equipment, and construction materials [7]. BPA is also widely used in the production of consumer goods [2]. The continuous release of BPA into environmental matrices such as air, water reservoirs, and soil, as well as the identification of this bisphenol in human samples [8], has prompted the imperative to implement preventive measures, including the use of its analogs, BPF and BPS. They take into account the fact that the production of BPA between 2022 and 2027 is projected to increase by 6% annually, with an estimated volume of 10.6 × 10^9^ kg in 2022 [9,10]. Additionally, more alarming data is available regarding global thermoplastic production. In 2025, this production will increase from 370 × 10^9^ kg to 445.25 × 10^9^ kg and is projected to reach around 590 × 10^9^ kg by 2050 [11]. This trend will also lead to a higher production of BPF and BPS, which are widely used in their synthesis.

The utilization of BPF as a monomer for polyamides, copolymers, and polycarbonate polyesters is just as significant as its role in producing epoxy resins that require enhanced strength and higher solid content [12,13]. On the other hand, BPS, in addition to its use as a corrosion inhibitor in epoxy adhesives and a wide range of synthetic materials such as dialyzers, dashboards, food packaging, receipts, and dental materials, has become an important substitute for BPA in thermal paper production. The consumption of this BPS-based product increased by 153% in 2019. The interest in BPF and BPS has led to their production at a level of 1 to 1–10 × 10^9^ kg per year [14].

The question about the safety of BPA substitutes arises from the structural similarity of these compounds and the proven equally toxic effects on the human body [15,16]. As a result, in 2017, BPA, BPF, and BPS were added to the Human Biomonitoring for Europe (HBM_4_EU) list, an initiative that assesses the health consequences for individuals exposed to bisphenols [17]. All three bisphenols were also included in the list of substances potentially disrupting the endocrine system (TEDX) [18]. Additionally, both BPA and BPS are part of the Community Rolling Action Plan (CoRAP) [14]. Bisphenols are also classified as ‘priority substances’ on the ATSDR’s Substances Priority List, highlighting their toxicity and prevalence in the environment [19].

Admittedly, knowledge about the toxicity of bisphenols for humans and animals, the degradation potential of microorganisms towards BPA, BPF, and BPS, as well as the frequency of their occurrence in environmental media is quite widespread. However, amidst these studies, there are no reports on the impact of the increasing pressure of the discussed bisphenols on the autochthonous soil microbiome, especially agricultural soils, determined based on the structural diversity of the microbiome and the number of microorganisms, as well as the response of soil enzymes. Both microorganisms and soil enzymes are recognized as reliable biological indicators, sensitive to environmental stress, and are referred to as critical parameters within the framework of simulating soil processes [20]. Therefore, a review of the existing research on these interactions, juxtaposed with a synthetic characterization of the effects of bisphenol penetration into major environmental media, is an essential step to shed light on the comparative scale of disruptions to soil equilibrium when exposed to BPA, BPF, and BPS. This will also allow for the systematization of knowledge about differences in the toxicity of BPA and its analogs, particularly within the soil environment, which undeniably affects crop yield. 

## 2. Chemical Characterization of Bisphenols

**Figure 1:**
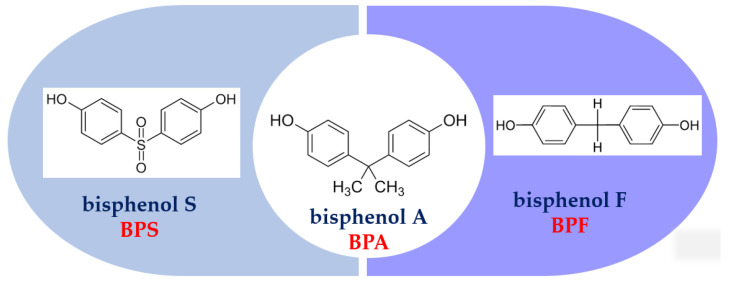
Chemical structure of BPA, BPF, and BPS.

The bisphenol synthesis process is not complicated. It involves the condensation reaction of phenol with appropriate solvents and catalysts. The synthesis of BPA occurs through the involvement of two phenol molecules and one molecule of acetone or hydrochloric acid. The reaction is catalyzed by ion-exchange resin [21]. In the case of BPF, formaldehyde serves as the solvent, and a Brönsted acidic ionic liquid acts as the catalyst [22]. BPS, on the other hand, is formed through the combination of phenol and sulfur trioxide [23]. BPF differs from BPA due to the absence of two methyl groups, whereas BPS has two phenolic functional groups positioned on either side of the sulfonic group. As a result, BPF exhibits lower polarity, and BPS showcases higher thermal stability [24,25]. The characterization of selected physicochemical properties of BPA (C_15_H_16_O_2_); BPF (C_13_H_12_O_2_) and BPS (C_12_H_10_O_4_S) (Figure 1) is presented in Table 1. 

The physicochemical properties of bisphenols that significantly determine their retention, transport, and fate in various environmental media include three partition coefficients: air-water (log*K*_AW_); octanol-air (log*K*_OA_); octanol-water (log*K*_OW_)_,_ and soil adsorption coefficient (log*K*_OC_), water solubility (*S*_W_) and vapor pressure (*V*_P_) [26]. The adsorption process of bisphenols in the soil is determined primarily by their hydrophobic properties [27]. 

**Table 1:** Selected physicochemical properties of BPA and related analogs.

Acronym	MW g mol^−1^	BCF ^a^	log*K*_AW_^b^	log*K*_OA_^b^	log*K*_OW_^b^	log*K*_OC_^b^	*S*_W_ ^b^ mg dm^−3^	*V*_P_ ^b^ (Pa)
BPA	228.29	71.85	−9.00	12.74	3.64	4.88	120	5.6 × 10^−6^
BPF	200.23	34.73	−9.70	12.58	3.10	4.47	408	1.2 × 10^−4^
BPS	250.27	36.97	−13.00	14.61	1.65	3.88	3518	6.4 × 10^−8^

MW—molecular weight; BCF—bioconcentration factor; partitioning coefficients: log*K*_AW_—air-water; log*K*_OA_—octanol-air; log*K*_OW_—octanol-water; log*K*_OC_—soil adsorption coefficient; *S*_W_—water solubility; *V*_P_—vapor pressure, a—Zaborowska et al. [28]; b—ChemSpider—Predicted Properties, EPISuite, KOAWIN v1.10 estimate, 25 deg. C [29].

## 3. Sources of Bisphenols in the Environment (Air, Water, Soil)

### 3.1. Air

Bisphenols can enter the air from anthropogenic sources, such as the thermal destruction of plastics, transport of recycling materials, and emissions from landfills and waste treatment plants [26]. Air pollution in economically developing countries is of particular concern, mainly due to the uncontrolled burning of e-waste [30]. Public concern has intensified over the past 10 years due to growing environmental awareness of the effects of their storage and transformation [26]. Morin et al. [31] pointed out the dominance of BPA in dust just after episodes of waste incineration. There is also much controversy over the burning of household waste in open drums, which generates BPA emission rates of 9.7 mg kg^−1^ of waste [32]. That open burning of waste is hazardous in its effects is also evidenced by Fu and Kawamera’s [33] report on BPA concentrations in southeast China (8200–14.500 pg m^−3^ of air) and India (average of 4550 pg m^−3^ of air). However, it is worth mentioning that due to atmospheric oxidation of the gaseous fraction of bisphenols by hydroxyl radicals, their airborne half-life is relatively short (0.11–3.62 days) [34]. Nonetheless, in the air, bisphenols are more hazardous in their monomeric form than in their polymeric form, especially when exposed to high temperatures or UV radiation [35]. Thermal degradation of BPA leads to the formation of 4-hydroxybenzoic acid and bis(4-hydroxyphenyl)methane [36]. At elevated temperatures, emissions occur through hydrolysis and breaking of carbonate bond chains [37]. Therefore, higher temperatures in manufacturing facilities promote greater emission of bisphenols into the atmosphere. Nevertheless, Graziani et al. [38] observed similar tendencies even during winter. These were likely induced by reduced photochemical reactions of bisphenol with hydroxyl radicals. According to the reports of Liao et al. [39], the total concentrations of bisphenols in indoor dust samples collected from the USA, China, Japan, and Korea ranged from 0.026–111 µg g^−1^ of dust, primarily dominated by BPA. Increasing concentrations of BPF and BPS in the air unequivocally indicate the implementation of policies aimed at minimizing the use of BPA and are indirectly related to the socio-economic status of developed countries. The obtained parameters presented in Table 2 were also directly influenced by limited room ventilation [26].

The significance of microplastics as carriers of bisphenols in the air is currently under discussion [41]. Non-covalent hydrogen and halogen bonds may contribute to adsorption onto microplastics [42]. Hydrogen bonds form between alkyl groups and aromatic rings, whereas halogen bonds are induced between π electrons of benzene rings and halogen atoms [43].

### 3.2. Water

The main sources of BPA, BPF, and BPS contamination of aquatic environments (freshwater, groundwater, drinking water) include manufacturing facilities synthesizing bisphenols, waste dumps, wastewater discharges, sewage treatment plants, and uncontrolled disposal of products containing these xenobiotics [2,44]. The extent of water pollution by bisphenols has been assessed so far through the prism of BPA, BPF, and BPS. The majority of reports pertain to their contamination of surface waters. Bisphenols have been identified in waters from regions including China, Poland, and Japan [45,46,47]. The presence of BPS in 16% of 282 tested samples of river sediments from the USA, Japan, and Republic of Korea was determined, reaching levels of up to 1970 ng g^−1^ d.w. of sediments [48]. Relatively significant pollution of BPA within the range of 4.62–29.92 µg dm^−3^ was also detected in river waters in Turkey [49]. The concentration of this bisphenol has also been tested in surface waters of Europe, in Spain, Germany [50,51], as well as Romania [52], and they amounted to 87–126; 28–560; and 22–135 ng BPA dm^−3^ of water, respectively. Lalwani et al. [53] also linked the highest concentrations of BPA (14,800 ng dm^−3^) in an Indian river to the discharge of untreated sewage from a treatment plant. Nevertheless, among a wide range of its substitutes, high concentrations of BPS and BPF were also detected in 53 samples taken from surface waters. In rivers of Southeast Asia, the concentrations of BPS and BPF were 24.8 and 9.0 ng dm^−3^ of river waters, respectively [54]. Sewage from the metropolitan area of Seoul generated water contamination in the lower part of the Han River at the level of 241 ng bisphenols dm^−3^ of water [55]. An important source of water contamination with phenolic compounds is also leachate from waste landfills. Corrales et al. [2] reported concentrations exceeding 17,000 µg BPA per dm^−3^ in their composition. Analyses of marine water samples clearly indicate increasing pollution not only with BPA but also with BPS. According to Xie et al. [47], BPS was the third dominant BPA analog in Chinese marine waters. In the Baltic Sea, BPS content was found to be similar to BPA [45]. In groundwater, the US Geological Survey classified BPA as one of the five most commonly occurring organic contaminants [56].

The solubility of BPA, BPF, and BPS in water is attributed to their hydrophilic properties [27]. In these ecosystems, they also undergo photodegradation, including photocatalysis or photooxidation, as well as adsorption onto suspensions [57,58,59]. Therefore, the half-life of bisphenols in water is determined by turbidity, turbulence, and pH. For BPA, it ranges from 66 to 160 days [60]. Intermediate products of BPA photolysis include 4-isopropylphenol and semiquinone derivatives of BPA [57]. Besides bioaccumulation, bisphenols also undergo biodegradation in water involving bacteria, fungi, and algae [61]. The most commonly identified bacterial metabolic intermediates are 4-hydroxyacetophenone (HAP) and hydroquinone (HQ) [60]. *Ulva prolifera*, representing algae, cultivated under continuous light conditions (12:12 h), in water contaminated with 100 µg BPA dm^−3^, was able to remove this phenolic compound at a level of 98.5% [61]. On the other hand, *Pleurotus ostreatus* eliminated BPA from water within 12 days. The key enzyme responsible for this process was laccase [62]. One of the latest techniques for removing bisphenol A and its analogs from water is the utilization of microbial fuel cells based on extracellular electron transfer by exoelectrogens [63].

### 3.3. Soil

Due to the permanently increasing dispersion of bisphenols in agriculturally managed soils, researchers are increasingly trying to figure out what their main sources are and the fate and persistence of both BPA and its substitutes BPF and BPS in these ecosystems. Currently, it has been proven that phenolic compounds penetrate soils along with herbicides [64,65,66] and insecticides [67,68]. Among significant herbicides, in addition to dichloro-diethyl-3-chloroethane and 2,4-dichlorophenoxyacetic acid, pentachlorophenol plays a major role. It was projected that the production of this compound would reach approximately 50 Mt in 2020 [66]. Also noteworthy is p-hydroxybenzonitrile (p-HBN), which is desirable during agrochemical treatments and is produced based on butylated hydroxytoluene (BHT) [69]. Besides pesticides, biosolids also serve as an important source of soil contamination with bisphenols [70]. It is worth emphasizing that both BPA, BPF, and BPS are recognized as the most persistent in sediments (t_1/2_ = 135–1621 days), less so in soils (t_1/2_ = 30–360 days) [1]. The content of BPA in sediments is in the range of 10–100,000 µg kg^−1^ d.m. of sediments. Significantly, the highest values of this bisphenol in sediments are generated in Europe (95,000 µg kg^−1^ d.m. of sediments), whereas lower levels are found in North America (about 14,200 µg kg^−1^ d.m. of sediments) [71]. Yu et al. [72] also estimated lower values of BPS in sediments compared to BPA, reaching a maximum of 1480 µg kg^−1^ d.m. In turn, Lee et al. [73] noted high concentrations of bisphenols (86.7–1780 µg kg^−1^ d.m. of sediments) in Korean sludge from domestic sewage treatment plants. BPA, BPF, and BPS were also dominant in sediments from sewage treatment plants in China and India [53,74]. This arguably provided the basis for demonstrating the highest levels of BPA in sewage sludge from these countries, reaching 2340 and 17,400 µg m^−3^ of sludge, respectively [75]. According to Pèrez et al. [70], an undeniable source of bisphenols in agricultural soils is the use of treated wastewater for filed irrigation [76]. The condition of the soil is also affected by electronic waste disposal facilities. This is evidenced by concentrations of BPA exceeding 100 µg kg^−1^ estimated in soil samples exposed to the effects of e-waste recycling [77]. 

After bisphenols enter soils, their persistence is primarily determined by the physicochemical properties of these phenolic compounds [78,79]. To understand the transformations that BPS undergoes in the soil, its fate was traced using ^14^C-ring-labeled BPS (^14^C-BPS) [80]. Cao et al. [80] proved that BPS was 53.6% mineralized. Unextractable residues resulting from the formation of ester bonds in soil matrices constituted 45.1%, with the content of the extractable form being around 3.7%. The re-mixing of unextractable residues of BPS with soil resulted in their instability and bioavailability, which in a way answers questions about the scale of toxicity of this bisphenol to soil ecosystems. BPS undergoes rapid dispersion in soil, with a half-life t_1/2_ = 2.8 days. In clay soil, the rate of this process was even higher (t_1/2_ < 1 day) [81]. Observations by Li et al. [82] suggest that in aerobic soils, BPS exhibited dispersion at a rate similar to BPA (0.8–7 days). The phenomenon of abiotic formation of unextractable residues also applies to BPF, both in aerobic and anaerobic soil conditions [83]. This process is induced by the adsorption of bisphenols onto soil matrices through the formation of organic-mineral complexes [84]. Therefore, an important role in the migration and transformation of this group of xenobiotics is also attributed to dissolved organic matter (DOM) [85]. Gan et al. [86] found that the sorption mechanism is based on the synergy of hydrophobic forces and hydrogen bonding, facilitated by functional groups such as –OH i –COOH. 

Among the most commonly employed methods for removing bisphenols from various ecosystems, including soils, efficient processes include oxidation, electrochemical methods, adsorption, photocatalysis, and ozonation. The potential of these methods corresponds to the properties of phenolic compounds. However, these methods generate hazardous metabolites and are associated with high energy consumption [87]. Therefore, significant importance is attributed to biocatalytic or enzymatic degradation as an alternative to physicochemical methods [88].

## 4. Microbiological and Biochemical Disturbances in Soils Exposed to Bisphenols

### 4.1. Microbiological Degradation of BPA, BPF, and BPS in the Soil Environment

Exploring the potential of microorganisms and their metabolic interactions with bisphenols based on metagenomic studies, including integrated transcriptome-metabolome analysis, offers an opportunity to comprehend biodegradation processes [89]. According to the International Patent Classification (IPC), these processes primarily involve bacteria. [90].

The degradation rate of bisphenols in soil is influenced not only by molecular characteristics and microbial diversity [87]. Biological, as well as physical and chemical properties of soils are responsible for it. This process is determined by a combination of factors including atmospheric oxygen availability, pH, temperature, humidity, the content of dissolved organic matter (DOM), the presence of manganese oxides, towards which BPS exhibits higher reactivity compared to BPA, iron oxides, and the oxidation of iodide to hypoiodous acid (HOI), which is significant in the transformation of bisphenols [91,92,93,94,95]. Enhanced biodegradation of BPA, BPF, and BPS is also associated with an increase in temperature to 15–30 °C [94]. This process occurs intensively when the C:N:P ratio is approximately 100:10:1 [96]. It should be emphasized that phenols and their derivatives are major building blocks of humic substances, exhibiting the highest affinity for humin fractions [97]. Therefore, dissolved organic matter, responsible for sorption, complexation, and ion exchange, triggers bisphenol degradation processes through the synergy of hydrophobic interactions and hydrogen bonding with soil mineral components [85,98]. The panacea for establishing a comparative scale of soil homeostasis disturbances exposed to the influence of BPA, BPF, and BPS lies in tracing the response of the soil microbiome to these xenobiotics, ranging from the biodegradative potential of microorganisms to their sensitivity to bisphenol pressure in soils. So far, 109 bacterial strains capable of degrading bisphenol A alone have been reported in the literature [8]. Among the microorganisms isolated from the soil, the main genera analyzed in terms of the biodegradation potential of BPA, BPF, and BPS have so far been *Pseudomonas*, *Sphingomonas*, *Bacillus*, and *Sphingobium* (Table 3).

The role of nitrifying bacteria should not be underestimated either, as they participate, along with *Pseudomonas* sp., in the cometabolism of BPS [114], as well as bacterial consortia such as *Pseudomonas umsongensis*, *Bacillus mycoides*, *Bacillus weihenstephanensis*, and *Bacillus subtilis*, which mitigate the negative effects of BPF [115] and BPS [116] soil contamination.

The positive reaction of the microbiome to bisphenols is closely integrated with their recognition by microorganisms as a source of carbon and energy [117]. It is attainable primarily due to microorganisms being equipped with an extensive pool of genes that induce enzymes participating in the catabolism of these organic compounds. Genes are usually located in chromosomes or degradation plasmids [118]. Among the most significant are the genes identified in microorganisms such as: *Pseudomonas umsongensis* (rftbADB, rfBC, genes encoding halohydrin dehalogenase (HHDH)) [119], *Bacillus* sp. (FabK) [120], *Acinetobacter calcoaceticus* (bamA, pheA2A1, PheR (AraC family), NCIMIB 8250), *Pseudomonas pseudoalcaligenes* (NCIMB 9867) [121], *Pseudomonas aeruginosa* JI104 (xyIEJI104), *Pseudomonas stutzeri* (nahH), *Pseudomonas* sp. PH11 (1,2-dioxygenase gene), *Pseudomonas* sp. PH7 (2,3-catechol dioxygenase gene) [122], *Corynebacterium glutamicum* (ncgl2587) [123], *Escherichia coli* BL21 (pET28a, bisdAB) [91] and cytochrome P450 monooxygenase (CYP) gene attributed to *Sphingomonas bisphenolicum* [124]. 

**Figure 2:**
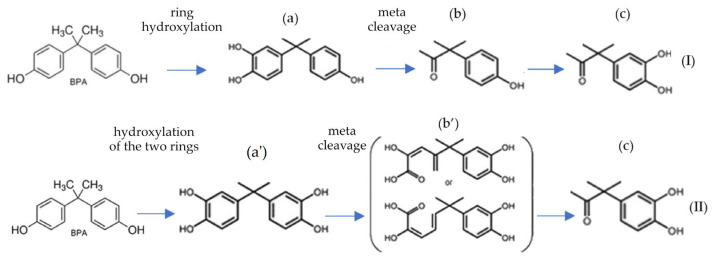
Two types of metabolic pathways for BPA degradation by *Sphingomonas fuliginis* IMO with hydroxylation of one (I) or two (II) aromatic rings; (**a**)—3-hydroxy BPA, (**b**)—3-(4-hydroxyphenyl)-3-methyl-2-butanone, (**c**)—3-(3,4-dihydroxyphenyl)-3-methyl-2-butanone; (**a’**)—2,2-bis(3,4-dihydroxyphenyl) propane, (**b’**)—ring cleavage location was not identified.

It has been demonstrated that microbial transformation strategies for BPA, BPF, and BPS are diverse [109,125,126], although a common initiating step is the presence of hydroxyl groups in the soil indicating the availability of oxygen as a co-substrate [127]. Two main degradation pathways for BPA, BPF, and BPS have also been characterized, involving important hydroquinone and p-benzoquinone metabolites, independent of the aromatic ring configuration. These pathways include hydroxylation of one or two aromatic rings and subsequent meta-cleavage of the molecule (1), followed by rearrangement of the aliphatic methyl group skeleton (2) (Figure 2) [109,128].

BPA can also be degraded by *Mycobacterium* sp. through a process of o-methylation to form monomethyl and dimethyl ethers [129]. When assessing the biodegradation potential of *Achromobacter xylosoxidans* B-16, three main intermediate products of BPA aerobic metabolism were identified: p-hydroxybenzaldehyde, p-hydroxybenzoic acid, and p-hydroquinone [111]. As for BPS, it undergoes rapid degradation through meta-cleavage and hydroxylation of the phenolic ring, leading to the formation of 4-hydroxy(methyl) benzenesulfinic acid. Eventually, BPS is mineralized to carbon dioxide and hydrogen peroxide [109]. Toyama et al. [110] proposed a degradation pathway for BPF involving rearrangement between its rings, resulting in the release of 1,4-hydroquinone, which undergoes partial mineralization and oxidation to 1,4-benzoquinone, as well as p-hydroxybenzoic acid that undergoes complete mineralization.

The full range of catabolic biochemical reactions initiating biodegradation can be used by microorganisms by equipping them with enzymes that catalyze these processes. Key enzymes include phenol monooxygenases (EC 1.14.13.7) and intradiol dioxygenases (EC 1.13.11.1) [130]. Dioxygenases belonging to the intradiol group play a crucial role in bisphenol degradation by introducing two oxygen atoms into the substrate. This leads to estradiol ring cleavage, generating intermediates for the Krebs cycle [131]. Among monooxygenases, significant roles are played by the cytochrome monooxygenase system, including cytochrome P-450, which catalyzes monomeric phenolic compound dimerization, ferredoxin, and ferredoxin reductase [132]. Additionally, the phenol hydroxylase, composed of six distinct subunits, is responsible for incorporating one oxygen atom into the aromatic ring. The toluene/o-xylene monooxygenase (TOMO) also participates in the hydroxylation of various positions on the aromatic ring [133]. The capability for bisphenol transformation is also possessed by extracellular laccases produced by bacteria of the *Pseudomonas* genus [134] and *Bacillus* [132], which catalyze oxidation, decarboxylation, and demethylation of bisphenols [125].

The biodegradation of bisphenols, including BPA, BPF, and BPS, is also carried out by mold fungi. Their potential in this regard is associated with the activity of laccases, as well as cytochrome P-450, manganese peroxidase (MnP), triphenylmethane reductase, lignin peroxidase (MnL), and polyketide synthase (PKS), which catalyze the degradation process of bisphenols [135,136]. With their participation, bisphenols are transformed into aromatic intermediates such as gentisic acid, gallic acid, protocatechuic acid, catechol, pyrogallol, hydroquinone, and hydroxyquinol, which ultimately transform to pyruvate or acetyl-CoA [126]. An example of an effective BPA degrader is *Chaetomium strumarium* G51, whose biodegradation potential is attributed to genes responsible for laccase synthesis [137]. *Penicillium chrysogenum* has been identified by Leitao et al. [138] as metabolites generated by molds relevant in biodegradation through para-hydroxylation, yielding hydroquinone further transformed into 1,2,4-trihydroxybenzene. Ultimately, this compound undergoes transformation into maleino-acetate. According to Shedbalkar et al. [139], *Penicillium ochrochloron* is also capable of catalyzing single-electron oxidation of bisphenols through lignin peroxidase synthesis. Among the pool of fungi significant in their biodegradation process are also *Aspergillus niger* equipped with polyketide synthase (PKS) responsible for the dimerization of monomeric phenolic compounds [140], and *Mucor mucedo* associated with triphenylmethane reductase, equally effective in bisphenol degradation. Noteworthy is the phenomenon of alcohol dehydrogenase accumulation dependent on NAD in response to oxidative stress observed in *Mucor plumbeus*, corresponding to the need for higher energy production levels under the pressure of phenolic compounds [117]. It is worth emphasizing the debated potential of the fungus from the genus *Podospora*, whose response to exposure to phenolic compounds is diverse. Despite being rich in three types of laccases—ABR1 oxidase, ascorbate oxidase (AO1), and ferroxidase (FET3)—it exhibits a subtle dependence [79,141]. This pertains to the high sensitivity of mold fungi of this genus to the phenolic intermediate, hydroquinone [142]. In the case of radiotrophic fungi, the biodegradative capacity towards bisphenols is also supported by a wide array of enzymes catalyzing the degradation processes of these compounds. Among them are proteases, cellulases, catalases, chitinases, amylases, and lectinases [143]. 

### 4.2. The Response of the Soil Microbiome to Soil Contamination with BPA, BPF, and BPS

There is considerably less information available in the literature regarding the toxicity of bisphenols towards soil microbiomes. However, it is known that phenolic compounds are adsorbed onto cell surfaces through hydrogen bonding, hydrophobic interactions, or electrostatic interactions [144], as a result of which bisphenols interfere with the metabolism of purines, pyrimidines, and phospholipid fatty acids (PLFAs) identified as acyl components of glycophospholipids and glycolipids [145]. Importantly, less reactive bisphenols can also damage the endoplasmic reticulum, mitochondria, and nucleus [146]. According to Rasheed et al. [147], the blocking and disruption of lipid synthesis result from the binding of bisphenols to the bacterial cell membrane through intercalation. Besides affecting membrane permeability, bisphenols significantly modulate sporulation and amino acid expression [145]. Through limiting interactions between fatty acid chains, exposure to phenolic compounds can lead to the inhibition of microbial respiration and growth, ultimately resulting in cell lysis [148].

The broad spectrum of interventions exhibited by BPA, BPF, and BPS, including their negative impacts, undoubtedly finds its reflection in the inhibition of proliferation and reduction in the population of individual microbial groups in the soil, depending on the level of contamination by these xenobiotics. Importantly, in the study by Zaborowska et al. [79], lower tolerance of *Pseudomonas* sp., *Arthrobacter* sp., organotrophic bacteria, and actinomycetes to exposure to 10 mg BPA kg^−1^ d.m. of soil compared to higher levels of contamination by this xenobiotic (100 and 1000 mg BPA kg^−1^ d.m. of soil) was observed. The obtained trends are complemented by the positive response of organotrophic bacteria, actinomycetes, oligotrophic bacteria, copiotrophic bacteria, nitrogen-immobilizing bacteria, ammonifying bacteria, *Pseudomonas* sp., and *Arthrobacter* sp. to the pressure of 100 mg BPA kg^−1^ d.m. of soil, except cellulolytic bacteria and *Azotobacter* sp. [28]. Furthermore, the application of 1000 mg BPA kg^−1^ d.m. of soil led to a spectacular 7-fold increase in the population of organotrophic bacteria, a 6-fold increase *in Arthrobacter* bacteria, a 3-fold increase in actinomycetes, and a 2-fold increase in cellulolytic bacteria compared to control subjects [149]. The highest toxicity of BPF to the soil microbiome is evidenced by its inhibitory effect already at the level of 100 mg bisphenol kg^−1^ d.m. of soil on the population of organotrophic bacteria, cellulolytic bacteria, copiotrophic bacteria, ammonifying bacteria, and *Arthrobacter* sp. Biotic stress associated with exposure to BPS led to the inhibition of the proliferation of *Arthrobacter* sp., cellulolytic bacteria, and copiotrophic bacteria, positioning it as the second most toxic compound in the series of bisphenols, following BPF and preceding the last in the series, BPA [28]. Although all bisphenols increased the growth rate of organotrophic bacteria and fungi as determined by the colony development (CD) coefficient, fungi proved to be the most sensitive to BPA, BPS, and BPF. Bisphenols significantly reduced the ecophysiological diversity of this group of microorganisms [28,79,149].

**Figure 3:**
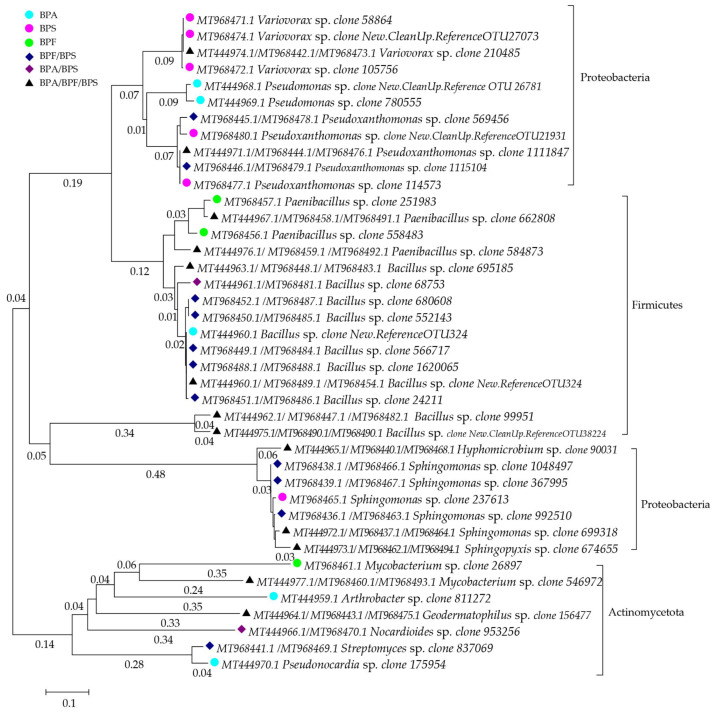
Phylogenetic tree of bacteria isolated from soils contaminated with BPA, BPF, and BPS using the neighbor-joining method. Original research by the authors [28].

The moderating force of bisphenol interactions is also evident in their influence on the genotypic and phenotypic diversity of the soil microbiome [28,79,150]. Amplicon sequencing of bacterial taxonomic groups based on the V3-V4 region of the 16S rRNA gene outlined changes in the taxonomic diversity of soil microorganisms subjected to the pressure of the three analyzed bisphenols, as presented in Figure 3 [79]. 

It was demonstrated that BPA reduces the abundance of *Actinobacteria* OTUs in favor of *Proteobacteria*, *Bacteroidetes*, and the phylum *Chloroflexi* [150]. On the other hand, BPS and BPF are capable of generating opposite reactions, increasing the abundance of *Actinobacteria* while having a negative impact on the abundance of *Proteobacteria* and *Acidobacteria*. In the study by Zaborowska et al. [28], this was reflected in the emergence of unique bacterial genera associated with individual bisphenols, as presented in Table 4.

The soil microbiome profile exposed to 1000 mg BPA kg^−1^ d.m. of soil was also shaped by bacteria from the genera: *Sphingomonas*, *Devosia* and *Achromobacter* [79], and *Novosphingobium*, *Luteibacter*, *Sphingobium*, *Chitinophaga*, *Mucilagnibacter* [149]. These microorganisms are capable of co-metabolizing or utilizing BPA as a carbon source. The negative impact of this phenolic compound was evident in a significant reduction in the abundance of OTUs belonging to *Cellulosimicrobium*, *Kaistobacter*, *Terracoccus*, and *Stenotrophomonas* [79]. 

The list of microorganisms sensitive to 1000 mg BPA kg^−1^ soil should also include mold fungi belonging to the class *Saccharomycetes*, whose OTU abundance in the soil contaminated with this xenobiotic decreased by 31.84% compared to the control. BPA also proved to be an inhibitor of the *Vishniacozyma* sp. population, reducing it from 67,180 OTUs to 2900 OTUs. Although in the BPA-contaminated soil, fungi belonging to the phylum *Ascomycota* accounted for as much as 96.70% [149]. Among its representatives, genera sensitive to this phenolic compound were identified: *Botryotrichum* sp., *Stachybotrys* sp., *Podospora* sp., *Minimedusa* sp., and *Solicoccozyma* sp. classified under the phylum *Basidiomycota*, as well as *Mortierella* sp. assigned to the phylum *Mortierellomycota* [79]. The differential effect of BPA was manifested in the stimulation of the proliferation of both fungi of the class *Eurotiomycetes*, whose OTU abundance increased by as much as 60.42% compared to the control objects, thanks to an intense increase in the abundance of fungi of the *Penicillium* genus: *Penicillium elleniae*, *Penicillium subrubescens* and *Penicillium javanicum*, as well as *Chrysosporium pseudomerdarium* [149]. According to Zaborowska et al. [79], when constituting a group of microorganisms with bioremediation potential against bisphenol A, the fungi *Iodophanus* sp., *Wrightoporia* sp. and *Tubulicrinis* sp. should also be included.

### 4.3. Sensitivity of Soil Enzymes to Soil Contamination with BPA, BPF, and BPS

The particular importance for determining the toxicological effects of BPA, BPF, and BPS on soil health is also attributed to the analysis of the interactions of these xenobiotics with soil enzymes, constitutive or induced typically by globular proteins, whose main role is releasing nutrient components into the soil environment [151,152,153]. However, these interferences have not been extensively studied thus far. Enzymes are responsible for maintaining a balance between the synthesis and degradation of organic matter, ensuring the stability of soil aggregates [154]. The dominant source of extracellular enzymes, aside from lysed microbial cells, includes enzyme complexes associated with organic matter, condensed tannins, or clay materials, stabilized through processes of adsorption or copolymerization. Therefore, their proteolysis and inactivation are significantly influenced by soil physical and chemical properties, such as texture and pH [155,156]. The significance of these parameters in the enzyme-bisphenol relationship is illustrated on the one hand by the influence of pH on altering the three-dimensional shape of the enzyme and its active site, and on the other hand, by the ability of bisphenols to exchange cations and exhibit increased sorption related to the content of exchangeable inorganic fractions, iron oxides, and clays [157,158]. The pool of enzymes whose activity is an important parameter of soil health includes dehydrogenases, catalase, *β*-glucosidase, urease, acid phosphatase, alkaline phosphatase, and arylsulfatase. Based on the sum of their activities, one of the most sensitive biochemical indicators of soil fertility (BA_21_) was constituted [159]. Therefore, analyzing the response of these enzymes to increasing bisphenol pressure is of great significance.

Enzymes whose response to bisphenols is not obvious, although they are considered reliable bioindicators of xenobiotic toxicity, are dehydrogenases [160]. These enzymes are closely dependent on the activity of living microorganisms. Their presence has been identified in polysomes, cell cytoplasm, bacterial spores, and fungal spores [155]. Hydroquinone is responsible for the toxicity of bisphenols toward dehydrogenases [161]. The inhibitory effect is exacerbated by the fact that the enzyme’s cofactor is pyrroloquinoline quinone (PQQ), which transports electrons from the substrate to ubiquinone in the oxidation process [162].

Research by Zaborowska et al. [163] proves that already 40 mg BPA kg^−1^ d.m. of soil is capable of inhibiting dehydrogenase activity by 30%, while 1000 mg BPA kg^−1^ d.m. of soil leads to a 50% inhibition. The inhibitory effect of BPF on this enzyme was much higher. A 60-day exposure to 5 mg BPF kg^−1^ d.m. of soil generated a decrease in dehydrogenase activity by 26% compared to uncontaminated objects. Under the pressure of 500 mg BPF kg^−1^ d.m. of soil, 7 times lower values of this parameter were found [115]. BPS should be considered an equally toxic analog of BPA towards dehydrogenases. Soil contamination with this xenobiotic at a level of 5 mg BPS kg^−1^ d.m. of soil significantly disrupted its homeostasis by reducing the activity of the discussed enzyme by 42.07%, and at a level of 500 mg BPS kg^−1^ d.m. of soil by 93.71% [116]. The mildest negative impact of BPA is also indicated by a twofold increase in dehydrogenase activity after the application of 0.1 mg BPA kg^−1^ d.m. to the soil [163]. The enzyme’s response is consistent with the findings of Dandzei et al. [164], which suggest that dehydrogenases are key biocatalysts in the hydrogenation of phenolic compounds. They participate in the conversion of ethylbenzene to 1-phenylethanol, and finally degrade acetophenone to benzaldehyde and benzoic acid.

The negative response of urease to BPA, BPF, and BPS can be argued similarly to dehydrogenases, with the toxic impact of intermediate metabolites of bisphenols, including e.g., benzoquinones, which inactivate urease by binding to the Sγ atom of the thiol group αCys3222. The strength of the degradative effect of catechol on urease is based on the covalent modification of αCys592 stimulated in two ways: by radical or nucleophilic addition. The αCys555 thiol group is responsible for covalent adducts with quinone [79,165]. Mustafa et al. [166] emphasize that the increased toxicity of organic compounds is conditioned by the presence of a methoxy or hydroxy group in the phenyl ring, whereas Pèrez et al. [70] attributed it to nitro groups. These interactions likely led to the inhibition of urease activity by 20% and 68% in soils contaminated with 40 mg [163] and 1000 mg BPA kg^−1^ d.m. of soil [79], respectively, compared to control objects. Both BPF and BPS proved to be stronger inhibitors of the analyzed enzyme compared to BPA, as indicated by the negative response of urease to increasing soil contamination with these phenolic compounds at a level of 100 mg kg^−1^ d.m. of soil, already after 15 days of exposure to the xenobiotics. A reduction in urease activity of 12.03% (BPA), 28.91% (BPF), and 32.89% (BPS) was observed at that time [28]. In turn, the pressure of 500 mg BPF and BPS generated lower enzyme activity by 58.63% [115] and 55.09% [116], respectively.

Disturbance of soil homeostasis may also be caused by inhibition of acid phosphatase and, to a lesser extent, alkaline phosphatase. Research proves that 1000 mg of BPA kg^−1^ d.m. of soil generates 16% lower acid phosphatase activity compared to the control, increasing the activity of alkaline phosphatase [149]. The greater sensitivity of acid phosphatase to BPS is evidenced by the inhibited activity of this enzyme after the application of 5 mg BPS kg^−1^ d.m. of soil (by 23%), whereas a similar reaction to this bisphenol was obtained against alkaline phosphatase in soil contaminated with 500 mg BPS kg^−1^ d.m. of soil [116]. In turn, the reaction of both enzymes to 500 mg of BPF kg^−1^ d.m. of soil was similar, equally negative, oscillating at the level of 40% inhibition of phosphatase activity. The negative response of phosphatases to bisphenols can be explained in two ways. Firstly, phosphatases are responsible for the phosphorylation of disodium phosphate leading to phenol synthesis, and the reaction of this compound with 4-aminoantipyrine in the soil generates quinone derivatives that are toxic to soil enzymes [167]. Secondly, it is associated with changes in the functional group structure of the enzyme due to the colocalization between the substrate and the enzyme on soil colloids [168]. It should also be emphasized that based on the response of both acid phosphatase and alkaline phosphatase to the pressure of BPA, BPF, and BPS, one can hypothesize that bisphenols at a dose of 100 mg kg^−1^ d.m. of soil stimulate the activity of these enzymes to varying degrees, except BPS against acid phosphatase. Interestingly, at such a dose, BPF enhanced the activity of alkaline phosphatase to the greatest extent, in contrast to BPA [28]. The stimulation of activity, especially alkaline phosphatase, can be attributed to the presence of carboxylic and hydroxyl groups in bisphenols, moderating the enzyme adsorption process on soil colloids, while disregarding the phenomenon of colocalization [169]. It should also be emphasized that phenolic compounds contribute to the increase in acid phosphatase activity due to their ability to lower soil pH, which affects the level of activators [170].

Biotic stress caused by soil contamination with BPA, BPF, and BPS positioned arylsulfatase, catalase, and *β*-glucosidase in a group of enzymes much less sensitive to bisphenols than dehydrogenases, urease, acid phosphatase, and alkaline phosphatase [28,79,115,149,163]. BPA at a level of 1000 mg kg^−1^ d.m. of soil increased the activity of arylsulfatase and catalase while inhibiting the activity of *β*-glucosidase [79]. However, in the studies by Zaborowska et al. [149], induction of *β*-glucosidase activity in response to that contamination level was observed. Similarly, the application of 800 mg BPA kg^−1^ d.m. of soil resulted in the stimulation of arylsulfatase and catalase activity and slight inhibition of *β*-glucosidase activity in the soil. Meanwhile, the pressure of 40 mg BPA induced a negative response in arylsulfatase and catalase, significantly disrupting the soil equilibrium [163]. Similarly, in soil contaminated with 50 mg BPF kg^−1^ d.m. of soil, the activity of the three mentioned enzymes was inhibited, whereas a soil contamination level of BPF that was 10 times higher enhanced their activity, except for *β*-glucosidase [115]. Analyzing the effect of BPS, it is important to highlight the negative enzyme response characterized by a 17.26% and 12.80% inhibition of *β*-glucosidase and arylsulfatase, respectively, except for catalase, whose activity increased by 13.45% compared to the control subjects [116]. In the study by Zaborowska et al. [28], exposure to 100 mg BPS also resulted in low sensitivity of *β*-glucosidase and arylsulfatase, as well as increased resistance of catalase to this xenobiotic. Soil contamination with the same amount of BPF revealed a much more toxic impact on the analyzed enzymes. When considering the negative response of *β*-glucosidase, it is important to take into account the significance of low activation energy (E_a_), values, the temperature coefficient (Q_10_), as well as kinetic parameters such as Michaelis–Menten constant (K_m_), and maximum velocity (V_max_) of the enzyme. They reduce its ability to adsorb at the hydroxyl phenol association site [168]. In turn, the increased resistance of catalase to phenolic compounds can be justified by its involvement in the decomposition of H_2_O_2_ responsible for oxidizing phenolic groups. This induces the peroxidation pathway [171]. It is also noteworthy that interactions between phenolic tyrosine (Tyr) residues and iron located in the catalase’s active center influence its preferences for peroxide-like compounds as substrates [172].

## 5. Reaction of Crop Plants to Soil Contamination with Bisphenols

Changes in the chemical stability within plant cells, catalyzed by glycosyltransferases (GT) and acyltransferases (AT), are attributed to a wide range of phenolic compounds, including phenolic acids, acetophenones, phenylpropanoids, naphthoquinones, stilbenes, flavonoids, and isoflavonoids [173], as well as the recently discovered five plant-derived bisphenols—capillarisenois A-E [174]. Schmidt and Schuphan [175] were among the first to demonstrate, through the distribution of ^14^C-labeled BPA in plant cell cultures, that BPA as a xenobiotic is rapidly absorbed and metabolized in winter wheat (*Triticum aestivum*) and soybean (*Glycine max*). This is ensured by the molecular weight of xenobiotics being less than 1000, enabling bisphenols to be easily taken up by plants from the soil, as well as the low values of the octanol-water partition coefficient (K_OA_). They guarantee greater availability of phenolic compounds from the air. Organic pollutants, including bisphenols, can be transported to plants through either the symplastic or apoplastic pathways [176]. The transformation of bisphenols in plants, resulting in the production of their metabolites, occurs through glucosylation, redox reactions, hydroxylation, and also glycosylation. This process leads to the formation of mono- and di-O-*β*-d-pyranosides, secondary metabolites that do not exhibit estrogenic activity [177]. Therefore, the toxicity of bisphenols to plants is a subject of debate.

Changes in root growth are often the most recognizable effects of bisphenol toxicity. However, as reported by Li et al. [178], doses below 3 mg dm^−3^ of water stimulate root cell elongation and proliferation exhibit effects similar to cytokinins and induce increased mitochondrial energy production [179]. According to Xiao et al. [180], morphological changes in roots become apparent after the application of more than 3 mg BPA kg^−1^ d.m. of soil. Bisphenols disrupt the structural integrity of cells by disrupting the microtubule matrix in meristematic cells at root tips, thereby reducing the capacity for mineral nutrient uptake. Microtubules can also become completely depolymerized [181]. Jadhav et al. [182] observed that a dose of 50 mg BPA kg^−1^ d.m. of soil enhances chromosomal aberration in the meristematic cells of plant roots, while 200 mg BPA disrupts the mitotic process of root cells. Under the pressure of bisphenols, there is also an increase in lipid peroxidation [183]. The response of cultivated plants to different levels of contamination and types of bisphenols is diverse. In the studies by Zaborowska et al. [79], 1000 mg BPA kg^−1^ d.m. of soil reduced the root mass of spring rapeseed (*Brassica napus*) to a much greater extent than that of maize (*Zea mays*) in relation to the aboveground parts. However, in both plants, stimulation of Ca^2+^ uptake and a decrease in Mg^2+^ content were observed. However, it should be emphasized that it has been demonstrated that the increase in Ca^2+^ in plants, resulting in its binding with calmodulin (CaM), is a plant response to biotic stress. Additionally, Ca^2+^ is indirectly responsible for the accumulation of phenol compounds in their aboveground parts through its involvement in the γ-aminobutyric acid (GABA) signal transduction pathway [184]. At the same level of soil contamination, the toxicity of BPA was also evident in the reduction of root biomass in sorghum (*Sorghum Moench*) and switchgrass (*Panicum virgatum*), as well as in the plant yield [149]. The moderating effect of increasing soil contamination with BPA on plants was demonstrated by the response of barley (*Hordeum vulgare*), where growth and development were stimulated after the application of 800 mg BPA kg^−1^ d.m. of soil. However, up to the level of 40 mg kg^−1^ d.m. of soil, bisphenol had a negative impact on this parameter [163].

The adverse effects of BPF on the yield of spring rapeseed are, however, much more pronounced than BPA [115]. The strength of BPF inhibition became evident even after soil contamination with 5 mg of bisphenol kg^−1^ d.m. of soil. Under the pressure of 500 mg BPF kg^−1^ d.m. of soil, disruptions in the growth of spring rapeseed occurred during germination, along with disturbances in the uptake of P, K, Mg, N, Ca, and K. This phenomenon results from the damage to plant cells at both structural and functional levels, stemming from the inhibition of catalase, ascorbate peroxidase, hexokinase, and phosphofructokinase activities [180]. These damages can also be caused by the activity of quinones, intermediate products of bisphenol degradation, which suppress the generation of reactive oxygen species (ROS), thereby closely correlating with the impairment of plant cell mitochondria [185]. The low Mg content in the presence of bisphenol also correlates with chlorophyll biosynthesis. The inhibition of this element uptake is associated with the suppression of gene expression coding for Mg-chelatase (ChII) and Mg-protoporphyrin methyltransferase [186]. The assessment of BPS toxicity towards *Brassica napus* highlighted its equally strong inhibitory effect, comparable to that of BPF [116]. While exposure to 500 mg BPS kg^−1^ d.m. of soil did not disrupt the germination process of *Brassica napus*, it hindered its growth at the stage of the third leaf development according to the BBCH scale. Moreover, similar to BPA and BPF, BPS reduced the biomass of the root more significantly than the above-ground parts of the plant [116]. The fact that bisphenols primarily accumulate in the roots of plants with high lipid content, and to a lesser extent in their stems and leaves, is justified by the synthesis of estrogens in soil contaminated with these xenobiotics. This could result from the interaction of bisphenols with sulfates and glucuronic acid, affecting the mobility of phenolic compounds between the soil and the plant [187]. A much more significant factor in moderating the process of morphological changes in plant roots is the level of abscisic acid (ABA) in the soil. Exposure to bisphenols results in an increase in ABA levels, thereby inhibiting the synthesis of endogenous hormones such as indole-3-acetic acid (IAA) and zeatin (ZT), which induce the growth of both roots and leaves [188]. Soil contamination with bisphenols also disrupts the balance between ethylene (ETH) and plant stress hormones (ETH/ZT, ETH/IAA), which also manifests in changes in the biological properties of plants [189]. It is not without significance that under the pressure of bisphenols, a deficiency of protein and amino acids is generated in the roots of plants, resulting from the inhibition of ammonia assimilation [190].

Bisphenols also interfere with the photosynthesis process occurring in plants. This phenomenon is diverse and dependent on individual plant preferences. In the study by Zaborowska et al. [79], BPA applied to the soil at a dose of 1000 mg kg^−1^ d.m. of soil did not significantly affect the chlorophyll content in the leaves of *Brassica napus* or *Zea mays*. However, in the leaves of *Sorghum Moench* and *Panicum virgatum*, an increase in chlorophyll content expressed by the SPAD greenness index was observed, with an increase of 60.73% and 44.52%, respectively, compared to control samples, in response to the pressure of 1000 mg BPA kg^−1^ d.m. of soil [149].

In turn, Kim et al. [191] noted a different reaction of *Vigna radiata* to 500 mg of BPA kg^−1^ d.m. of soil. Researchers postulate that this is a consequence of a reduction in the size of stomatal apparatuses. However, the toxic properties of bisphenols also lead to the disruption of fluorescence and a decrease in chlorophyll a and chlorophyll b in the plant.

Plants also evoke a wide range of defense mechanisms against the toxicity of bisphenols. One of these mechanisms is the regulation of gene expression, including detoxification genes such as glutathione transferase [192]. Plant resistance to bisphenols corresponds to their possession of cytochrome P450 monooxygenase (CYP8D1) located in the cytosol. This enzyme catalyzes the conversion and degradation of organic pollutants, including bisphenols, in the plant [164]. Preisner et al. [193] contest that dehydrogenases also play a significant defensive role. Their participation in the catalysis of NAD+/NADP+ electron acceptor reduction results in the extracellular oxidation of toxic bisphenol metabolites. Yang et al. [194] report that in the case of *Zea mays*, genes involved in flavonoid biosynthesis (C2 and FLS1), phenylpropanoid biosynthesis (4CL3 and PAL9), and six lignin-related genes contribute to increased resistance to bisphenols, including germination and response to xenobiotic stress. Importantly, the adaptive response of plants to bisphenol pressure involves the accumulation of polyphenols in their tissues. These are synthesized through two pathways: acetate/malonate polyketide and shikimate/phenylpropanoid [195]. The role of microorganisms should also not be underestimated, as plants serve as a reservoir for them as well. The biodegradation of bisphenols can occur through horizontal transfer of genetic determinants between the plant and its microbiome, equipped with the necessary genes to induce this process [196]. 

## 6. Toxicity of Bisphenols for Humans and Animals

Concerns regarding the harmful effects of bisphenols on human organisms, supported by in vitro research results, have prompted changes in decisions regarding the legally established status of these xenobiotics. They are part of well-considered economic strategies. These concerns are primarily associated with BPA. In 2006, the European Food Safety Authority (EFSA) deemed a daily intake of BPA at the level of 50 µg kg^−1^ as acceptable. Currently, this limit has been reduced to 4 µg kg^−1^ of body weight [197]. In 2017, the European Commission decreed to limit the use of BPA in thermal paper production to 0.2%, and this measure has been in practice since 2020. In 2018, restrictions on the use of BPA in can coatings were also introduced [198]. Awareness of the similarly high toxicity of BPF and BPS should be continuously deepened, as scientific studies have shown that these phenolic compounds are not significantly less toxic to human and animal organisms [199,200]. Researchers have pointed out their adverse effects on the nervous and immune systems, also documenting their cytotoxicity and genotoxicity. However, it should be noted that the polarity of their molecules contributes to the scale of bisphenol toxicity [201].

One of the manifestations of the harmful effects of BPA, BPF, and BPS is their interference with the functioning of the hormonal system [202,203]. As early as 1930, BPA aspired to be a synthetic estrogen [204]. The interference of BPA with the hormonal system is based on its interaction with various receptors, including the aryl hydrocarbon receptor (AHR), peroxisome proliferator-activated receptor (PPAR) [202], as well as estrogen receptors (ERα, ERβ, and γ(ERR-γ)) [205]. Nevertheless, it has been demonstrated that BPF exhibits 5 times higher estrogenicity than BPA, as verified in cell proliferation tests using human MCF-7 and HepG2 cells [203]. In turn, BPS not only induced rapid non-nomic signaling responsible for the proper pituitary cell response to estrogen but also disrupted the initiation of estradiol signaling, consequently enhancing prolactin release [206]. An important consequence of bisphenol toxicity is developmental disorders of the brain and nervous system. This is a result of the interference of phenolic compounds in two crucial processes: synaptogenesis and neurogenesis [207,208]. The neurotoxicity of BPF and BPS is manifested by the increased expression of 24 out of 84 genes related to dopamine and serotonin. Furthermore, similar to BPF, BPS also exerts inhibitory effects on 5α-reductase, a key enzyme responsible for neurosteroidogenesis in mammalian brains [209]. Research by Perera et al. [210] has also revealed a correlation between BPA exposure and depressive states and anxiety disorders in children.

Reports concerning the toxic impact of bisphenols on fetal development focus mainly on BPA [211]. Following exposure to this phenolic compound, an accumulation of 273 ng BPA g^−1^ in placental tissue was observed [212]. In humans, BPA, BPF, and BPS are metabolized into glucuronide and, to a lesser extent, sulfate conjugates. The rates of sulfation for all bisphenols are comparable. Sulfation represents an important metabolic pathway for the developing fetus [16]. Exposure to BPA during the perinatal period also increases the incidence of asthma and eosinophilic bronchitis in children [213]. Endometrial proliferation and miscarriage should also be added to the list of adverse reproductive effects associated with BPA [214]. Recent studies confirm that exposure to BPA and BPS, except for BPF, is undeniably associated with infertility [215].

The most spectacular consequence of the effects of bisphenols on the human body is the induction of cancerous changes. BPA is responsible for prostate cancer by disrupting AR-T877A mitogenesis in its cells [216]. Regulation of protein kinase signaling ½(MARK/ERJ1/2) inappropriately stimulated by BPA, leads to cancerous changes in ovarian and breast cells [217]. This bisphenol also disrupts oxidative phosphorylation in liver mitochondria [218]. In turn, BPS reveals its cytotoxicity in adrenal cortex cells (H295R), as well as hepatocytes and kidney cells [219,220]. Similarly, BPF focuses on inducing renal and hepatic carcinogenic changes by lowering the biosynthesis of both glutathione synthetase and glutaminase, which characterize the metabolome and lipidome of these organs [199]. Recent studies also indicate that exposure to BPS not only results in stomach cancer but also increases the proliferation and migration of its cells [221]. Bisphenols also manifest their toxicity among aquatic organisms. BPA, BPF, and BPS induce an increase in the activity of the CYP19a1 gene, involved in the transformation of androgens into estrogens in zebrafish [222]. They also lead to the phenomenon of fish feminization [223].

## 7. Conclusions

The dispersion of BPA in the environment associated with its increasing production is a consequence of its capability as a monomer to delay the oxidative degradation of synthetic materials. Due to these predispositions, BPA has become an indispensable component in electrical and electronic devices, a wide range of consumer goods, safety equipment, and construction materials. The increasing awareness of its penetration into ecosystems, including soils, waters, and air, and its negative impact on their functioning, has led to the replacement of BPA with its analogs, BPF and BPS, potentially less toxic. However, it still arouses many controversies and remains undetermined. The scale of toxicity has been precisely diagnosed mainly in humans and animals. The biodegradability potential of BPA, BPF, and BPS by both bacteria and mold fungi is also well recognized. Less attention has been given so far to the response of the autochthonous microbiome to the increasing pressure of bisphenols, manifested by changes in its structural diversity and microbial abundance, as well as the activity of enzymes, which are essential biological parameters for assessing soil health, including agriculturally used soils. The consequences of biotic stress related to the diverse toxicity of bisphenols position BPF as the most toxic, followed by BPS, with BPA being the least toxic phenol in the series for soil microorganisms. Considering the strength of the negative impact of bisphenols on the activity of specific soil enzymes, they can be ranked as follows: BPS > BPF > BPA. Initial reports indicate comparable or even greater toxicity of BPF and BPS not only towards soil microbiomes and their biochemical activity but also towards their stronger inhibitory effect on the growth and development of cultivated plants, which undoubtedly amplifies negative societal concerns. On the other hand, it encourages the search for effective solutions to eliminate the increasing environmental contamination by these xenobiotics.

## Figures and Tables

**Table 2:** Global levels of BPA, BPF, and BPS (pg g^−1^ of dust) indoors [40].

Location	BPA	BPF	BPS	Sample Collection (Year)
Europe				
Iasi, Romania	680	41	380	2012
Athens, Komotini, Erateini, Greece	1700	5500	1500	2014
Asia				
Patna, India	360	29	<12	2014
Jeddah, Saudi Arabia	1100	160	110	2013
Ansan, Anyang, South Korea	1100	1300	10	2014
North America				
Albany, United States	3800	4400	2.1	2014
South America				
Cartagena, Columbia	420	69	3.7	2014

**Table 3:** Selected microorganisms with biodegradative potential towards BPA, BPF, and BPS.

Microorganisms	Kind of BP	References
Genus: *Pseudomonas*		
*Pseudomonas putida* YC-AE1	BPA, BPS, BPF	[91]
*Pseudomonas aeruginosa* Gb30	BPA	[99]
*Pseudomonas putida* G320	BPA	[100]
*Pseudomonas* sp. BG-12	BPA	[101]
*Pseudomonas palleroniana* GBPI_508	BPA	[102]
*Pseudomonas* sp. ZH-FAD	BPF	[103]
Genus: *Sphingomonas* *Sphingomonas* sp. SO11	BPA	[104]
*Sphingomonas* sp. SO1a	BPA	[104]
*Sphingomonas* sp. SO4a	BPA	[104]
*Sphingomonas bisphenolicum* AO1	BPA	[105]
Genus: *Bacillus*		
*Bacillus* sp. YA27	BPA	[104]
*Bacillus megaterium* ISO-2	BPA	[106]
*Bacillus amyloliquefaciens*	BPA, BPF	[107]
Genus: *Sphingobium*		
*Sphingobium fuliginis* TIK1	BPA, BPS, BPF	[108]
*Sphingobium* sp. IT4	BPA, BPS, BPF	[108]
*Sphingobium fuliginis* OMI	BPA, BPS, BPF	[109]
*Sphingobium yanoikuyae* TYF-1	BPA, BPF	[110]
*Sphingobium yanoikuyae* FM-2	BPF	[110]
other bacterial species:		
*Achromobacter xylosoxidans* B-16	BPA	[111]
*Arthrobacter* sp. YC-RL1	BPA	[111]
*Klebsiella pneumoniae* J2	BPA	[112]
*Enterobacter asburiae* L4	BPA	[112]
*Cupriavidus basilensis* SBUG	BPA, BPF	[113]

**Table 4:** Unique bacterial genera isolated from soil contaminated with BPA, BPF, and BPS.

BPA	BPF	BPS
*Lysobacter*	*Caldilinea*	*Dactylosporangium*
*Steroidobacter*	*Arthrobacter*	*Geodermatophilus*
*Variovorax*	*Cellulosimicrobium*	*Sphingopyxis*
*Mycoplana*	*Prominomonospora*	

## Data Availability

Data are available by contacting the authors.
